# Overcrowding in the Nursing Profession—A Remedy

**Published:** 1899-09

**Authors:** 


### OVERCROWDING IN THE NURSING PROFESSION—A REMEDY.

THE profession of trained nursing is scarcely out of its teens and yet one already hears the cry that the field is overcrowded, a cry which the city physician, besieged by nurses in search of work, knows is only too true. Various remedies are proposed to relieve the condition for which the rapid increase in the number of hospitals is responsible, an increase which has worked ill to the physician as well as to the nurse.

A reduction in the number of training schools by the union of those connected with small hospitals will be a wise change, both because it will lessen the supply of nurses and because it will afford better instruction to those who now graduate from special hospitals with but inadequate training. The lengthening of the course of training to three instead of two years is also desirable. So, too, would be the change from a school in which the pupil nurse is paid for her work to one in which she pays for her tuition.

Yet, with all these checks to lessen the number of trained nurses, the profession will remain overcrowded so long as graduate nurses remain in the city and demand $20.00 a week for their services. There is, however, an almost limitless field for those women willing to go into the country and take for their services what their patients can afford to pay. To the woman who in addition to earning her living would like to feel that she is doing some good in the world the country town ought especially to appeal. The need for trained nursing there is great and the demand for the work will grow as its worth becomes known.

A physician in a village of 1,000 inhabitants gets from one-third to one half the fee for a given service which the city physician, practising among the well-to-do, receives. The nurse in a country town of this size could command from $7.00 to $10 a week. Living expenses will be much lower than in the city, and the diminished income will be more than offset by the lessened outgo. The social position of a nurse in a small town may, and usually will, be superior to that which she holds in the city where nurses live almost as a class apart.

It was the fortune of the writer not long ago to be called to the sick bed of a friend in a country town remote from the city. The attempt was made to find a nurse to relieve the tired-out sister who had acted as such. The only women in the village who did nursing, neither of whom could be had, were two, who during the lulls in business did washing and scrubbing. As nurses they received $1 a day. To those who are accustomed to the tender ministrations of the trained nurse, a sponge bath given by their laundress would at least seem rather unseemly. This town supported four physicians and the one in charge of the case said that he alone could much of the time keep one nurse employed

The woman to succeed in the country, and success surely awaits the right woman, must be one possessed of great adaptability, of good judgment, able to hold her tongue, willing sometimes to do work outside the usual line, and above all a woman of common sense. The country doctor will welcome her coming, for he will recognise in her an ally, for want of whom he has fought many a losing battle.

---

THE christian science question which was before the aldermen when the last issue of the JOURNAL went to press is still before the board, having at the last meeting before the August vacation been laid over until the first meeting in September by resolution introduced by the joker of the board of aldermen, John P. Sullivan. This may have been one of Alderman Sullivan's jokes. Maybe, too, it was the result of political friendship on the part of the alderman for the able counsel of the christian scientists, Mr. John Cuneen, who, though he is fighting under the green flag of fanatacism, is nevertheless fighting as he always fights, stubbornly and to the finish. The mere laying over of the proposed ordinance for thirty days will not affect the ultimate result one iota. It merely serves to give the scientists thirty days more in which to spread disease and violate the law. They do both with equal disregard for the discipline of civilisation.

It will be interesting now to watch the next meeting of the board of aldermen for two reasons: First, because vital public interests are at stake; secondly, because there is a growing suspicion that the game of politics is entering into the question. The city will elect aldermen this Fall.

---

THE "scientists" had a sort of a celebration in Jersey City a few days ago, and wound up with an experience meeting which must have been, in its entirety, one of the most remarkable affairs on record. Biblical miracles were discounted and thrown utterly in the shade. Besides the wonders performed by these latter-day saints the miracles, has reported in the Bible, were merely experiments and rather crude efforts at that.

According to the experience of those who had been saved all degrees of suffering and cured of all sorts of terrible maladies, the pastor of the church—the chief miracle-worker—had only to raise his hands above his head, and, lo! cancers shrivelled up and disappeared, epilepsy ceased from troubling, and the choreaics were at rest.

Maybe it would be more satisfactory to JOURNAL readers to have presented in narrative form a few of the "most glorious victories over matter." One woman rehearsed the old familiar tale of cancer cure; another believer had had rheumatism banished by a single prayer; another woman said her daughter had suffered with epilepsy for four years, and had "been made worse by doctors." "Four weeks ago," she continued, the pastor has prayed over the child, "and she has not had so many attacks since."

The sensation of the meeting came at the end of the experiences, as a sort of an hair-raiser for the wavering ones. An old woman, prefacing her remarks with a statement of her undying belief and faith in the efficacy of prayer, said she knew a man in Texas who had spilled acid in his eyes. The eye-balls were so badly injured that both were removed by surgeons. He became a believer and by the means of prayer, new eyeballs grew into the empty sockets, and now the man can see as well as anyone.

And these are the people who do not believe in disease! Is it any wonder they fight for delay in any movement which will bring them within reach of the law?

---

THE *New York Medical Journal* has recently called attention to a very careless habit of a large number of medical editors in failing to give credit for items of news and other paragraphs quoted from exchanges. We, ourselves, are often thus victimised but had concluded it was one of those conditions to be endured. It is possible, however, that it may not be amiss to invite attention to the subject and it cannot be done in any more effectual manner than to print what our distinguished contemporary says :

THE UNACKNOWLEDGED CLIPPING HABIT.

We must again call attention to the fact that we are dropping from our exchange list journals which we detect in wholesale clipping, without acknowledgment, from our columns. Accidents, of course, will occasionally occur, but instances are often so flagrant, as in the case of one journal which we have dropped this month for five such offenses in one number, that they can only be intentional. We are interested in the maintenance of that system of journalistic courtesy which is willing to place the free use of its material for the public good in the hands of editorial confréres. But that courtesy, like all others, deserves the acknowledgment which we ourselves consistently endeavor to give, and which we certainly expect.

THE word celiotomy which is gradually creeping into journalistic literature, is such a mongrel in derivation and meaning that we are surprised at its considerable use among lettered men. We have intended for some time past to invite attention to the undesirability of the word. The *New York Medical Journal*, however, whose editor is one of the most distinguished medical philologists, has taken up the question in a recent issue from which we quote :

"ABDOMINAL CELIOTOMY."

A few years ago the word celiotomy, to denote abdominal section, was brought forward with great insistence on the part of its advocates that it was a better word for the purpose than laparatomy. We have never thought so, and the word does not seem to be universally recognised as fully signifying abdominal section, for it is getting to be more and more common to see the expression "abdominal celiotomy" in print.

The word celiotomy, itself, is bad enough, but the terms "abdominal celiotomy" and "vaginal celiotomy" are an abomination. We print them when authors write them in original communications, for then there is no time to enter into an argument over the matter, but in the editorial pages of this journal they have always been excluded and they always will be.

---

THE State Board of Health of Kentucky has given notice that it will hereafter refuse to recognise as a basis for certificates to practise medicine diplomas from any medical college which does not in good faith comply with the requirements of the American Medical College Association, the American Institute of Homeopathy, and the American Eclectic Medical College Association, respectively, both as to preliminary education and four years' course of study. This means that no school that graduates three-year students will be recognised in Kentucky hereafter. The Board provided an examination for three-year graduates of the present year, as many of the students had attended such schools in ignorance of its advanced requirements, but found this course unsatisfactory, a large percentage of the examinations indicating incomplete preliminary education as well as imperfect medical training. This standard for the State of Kentucky was made and promulgated in 1891, to take effect the present year, but is again published that schools patronised by Kentucky students and future graduates expecting to practise in the State may fully understand the requirements of the board.—*Medical News*.

DURING the hearing before the board of aldermen on Tuesday, August 1, 1899, relating to the application of the health commissioner, Dr. Wende, for an amendment to the ordinances to compel christian scientists to report contagious diseases, the Hon. Tracy C. Becker, attorney for the Medical Society of the County of Erie, spoke in part as follows :

There are some very great misunderstandings in the minds of the public and of the opponents of this ordinance as to its object and as to the attitude of the physicians of the county toward it. There is an ordinance which provides that physicians shall report to the health department diseases which are contagious or infectious. There is a boundary surrounding the occult and mysterious which is not to be passed by any finite beings, and it may be that these people may be able to exercise powers that produce great results. The physicians of the Erie County Medical Society do not deny or affirm the efficiency of christian science, but they do say that if they are required to report cases of contagious disease, others shall do the same.

This is a succinct and able statement of the case from a medical viewpoint, and could cause no offense to any right-minded citizen.

